# Impact of an emergency department bypass referral pathway for surgically managed Type A aortic dissection diagnosed at non-aortic centers

**DOI:** 10.3389/fpubh.2025.1717962

**Published:** 2026-03-17

**Authors:** Zhixiang Cai, Tao Yan, Hulin Wang, Jinxia Zhang, Weida Zhang, Juncan Zhuang, Ben Zhang, Xianyue Wang

**Affiliations:** 1 School of Traditional Chinese Medicine, Hunan University of Medicine, Huaihua, China; 2 General Hospital of Southern Theater Command, PLA (People’s Liberation Army), Guangzhou, China; 3 The 925th Hospital of Chinese PLA Joint Logistics Support Force, Guiyang, China; 4 Department of Emergency Medicine, Jiangmen People’s Hospital, Jiangmen, China

**Keywords:** Type A aortic dissection, TAAD diagnosed at non-aortic centers, direct bypass protocol, emergency transfer pathway, surgical outcomes

## Abstract

**Objective:**

Surgical outcomes for Type A aortic dissection (TAAD) are significantly improved at high-volume aortic centers. Notably, approximately 60% of TAAD cases treated at these centers are referrals from outside hospitals, with nearly half presenting in an unstable condition upon arrival. To address critical delays in this population, our institution, in collaboration with the Chest Pain Center, implemented a regional *Direct Bypass Protocol* (DBP) in April 2019. This 24/7 pathway allows patients diagnosed with acute aortic syndrome at non-aortic centers to bypass the emergency department (ED) and proceed directly to surgical care. This study evaluates the impact of the DBP on surgical outcomes for TAAD patients diagnosed at non-aortic centers.

**Methods:**

We retrospectively analyzed medical records of TAAD patients diagnosed at non-aortic centers and transferred to our hospital for surgery between January 2018 and December 2023. Clinical outcomes before and after DBP implementation were compared.

**Results:**

The study included 144 patients in the Routine Referral Group and 149 in the Emergency Bypass Group. The two groups were well matched for demographics and comorbidities. Compared to the Routine Referral Group, the Emergency Bypass Group had a significantly shorter time from hospital arrival to surgery (8 h vs. 4 h, *p* < 0.001) and a higher rate of total arch replacement procedures (84.0% vs. 91.3%, *p* = 0.059). In-hospital mortality was lower in the Emergency Bypass Group (18.8% vs. 10.7%, *p* = 0.053). After propensity score matching, both 30-day mortality (12.5% vs. 4.5%, *p* = 0.031) and in-hospital mortality (15.2% vs. 7.1%, *p* = 0.056) were significantly reduced in the Emergency Bypass Group. The median hospital stay was also shorter post-matching (16 days vs. 17 days, *p* = 0.003). There was no significant difference in postoperative complication rates between the groups. Cox regression analysis showed that implementation of the DBP was associated with a reduced risk of mortality (*p* = 0.002, hazard ratio = 4.546, 95% CI: 1.721–12.004).

**Conclusion:**

A coordinated, point-to-point referral model between the Chest Pain Center and the Aortic Center integrating streamlined pre-hospital triage and a dedicated aortic surgical team significantly improves outcomes for TAAD patients diagnosed at non-aortic centers.

## Introduction

The natural mortality rate of TAAD is exceptionally high (1). Recent studies have shown that patients with TAAD who do not undergo surgical intervention face an hourly mortality increase of 2.6%, with the risk of death reaching 50% within 24 h of symptom onset (2). Moreover, over half of TAAD patients are already critically ill or hemodynamically unstable by the time they undergo surgery (3, 4). Prompt diagnosis and timely surgical repair specifically resection of the entry tear and restoration of true lumen flow are therefore essential to prevent fatal complications such as malperfusion syndromes and aortic rupture (5, 6).

In China, however, multiple systemic issues contribute to delays in surgical intervention. These include inadequate coordination between primary hospitals and specialized aortic centers, poor collaboration among emergency, imaging, and surgical departments, frequent misdiagnosis due to atypical presentations, inefficient referral processes, prolonged emergency department stays, and slow mobilization of surgical teams (7). Collectively, these factors result in a significantly longer time from symptom onset to definitive surgical treatment compared to international benchmarks. For example, the 2011 International Registry of Aortic Dissection (IRAD) reported a median time of 1.5 h from symptom onset to hospital admission and 4.3 h from diagnosis to surgery. In contrast, data from Fuwai Hospital in 2023 indicated a median time of 10.65 h from onset to admission and 13 h from emergency department arrival to surgery (8).

Delays in surgical treatment significantly increase the risk of aortic-related hemorrhagic and ischemic mortality, particularly among critically ill TAAD patients. Importantly, pre-operative deaths are often underreported in the literature (8). This problem is exacerbated by the uneven distribution of TAAD treatment resources across China. As of 2021, only 1,651 tertiary hospitals existed nationwide, accounting for just 4.51% of all medical institutions. In 2017, only 52 hospitals performed TAAD surgeries (9), and currently, fewer than 150 are equipped to do so (10). Additionally, China lacks a mature and efficient referral system for TAAD, often requiring multiple inter-hospital transfers before reaching a capable facility (11). Both pre-hospital referral delays and in-hospital treatment inefficiencies contribute to postponed surgical intervention and worsened patient outcomes. Recent literature reports that such delays double the overall mortality rate in TAAD patients (12).

Given the complexity and high risk associated with TAAD surgery, surgical outcomes are strongly correlated with aortic center volume (13). In the United States, more than 60% of TAAD patients treated at high-volume centers defined as those performing over 100 open aortic procedures annually are referred from external hospitals (14). Studies have shown that regionalized aortic center networks significantly reduce the time from diagnosis to surgery and are associated with lower mortality rates (15). For instance (16), the establishment of a specialist aortic on-call dissection rota service in Liverpool led to a reduction in mortality from 30 to 13.3% (*p* = 0.004). In Europe, a rapid referral network involving multiple hospitals within a defined geographic radius, combined with a 24/7 standby aortic surgical team, has been implemented to expedite patient transfers (17). This system achieved significantly lower in-hospital mortality (24.2% vs. 11.11%, *p* = 0.015) and 30-day mortality (20.3% vs. 8.08%, *p* = 0.010), with propensity-matched analysis confirming a substantial reduction in 30-day mortality (25.5% vs. 6.4%, *p* = 0.011).

Recognizing the significant benefits of a point-to-point emergency department bypass model for externally diagnosed TAAD particularly its ability to dramatically reduce in-hospital delays in specialist response after patient arrival our center launched a 24/7 acute aortic syndrome service in 2019. This program collaborates with more than ten nearby chest pain centers that lack TAAD surgical capabilities. The present study aims to assess the impact of this service model (Emergency Bypass Group) compared with the conventional referral pathway (Routine Referral Group) on surgical outcomes in patients with TAAD.

## Methods

The study was conducted in accordance with the Declaration of Helsinki and its subsequent amendments. The study was approved by institutional/regional/national ethics/committee/ethics board of the General Hospital of the Southern Theater Command (NZLLKZ2025092) and individual consent for this retrospective analysis was waived.

### Clinical data collection

A retrospective analysis was performed on medical records of TAAD patients who were externally diagnosed and subsequently transferred to our center for surgery between January 2018 and December 2023. All patients included in this study were preoperatively diagnosed via external computed tomography angiography (CTA). Patients were categorized based on their transfer pathway: 1. Emergency Bypass Group: Point-to-point emergency department bypass transfer. 2. Routine Referral Group: Conventional transfer via the emergency department.

### Inclusion criteria

Emergency Bypass Group: Patients transferred after April 2019 using the dedicated point-to-point bypass pathway (bypassing the emergency department). Data for this group were prospectively collected and recorded in an electronic database by the surgical team.

Routine Referral Group: Patients with externally diagnosed TAAD transferred via the conventional pathway (through our emergency department, followed by consultation, transfer to the specialty unit, and then surgery) between January 2018 and December 2023.

### Exclusion criteria

Patients meeting any of the following criteria were excluded:

1. Subacute or chronic aortic dissection. Refusal of treatment due to religious beliefs or delays in transfer/surgery attributable to such refusal.
2. Diagnosis of other acute aortic syndromes (e.g., penetrating atherosclerotic ulcer) initially managed as an emergency TAAD transfer.
3. Patients undergoing emergency surgery involving only descending or abdominal aortic segments.
4. Patients diagnosed with TAAD but not managed as a surgical emergency, including those with surgical indications who opted for conservative management or were discharged against medical advice.
5. Patients who did not undergo surgery due to death or discharge against medical advice after departure from the transferring hospital but before admission to our unit/hospital or before surgery (Excluded to specifically assess the impact of the transfer model on patients who actually received surgical intervention, minimizing survivor bias).
6. TAAD patients transferred from outside Guangdong Province or with a single journey of more than 3 h.
7. Patients primarily diagnosed or initially presenting at our hospital.

### Indicators of observation

1. Preoperative clinical data: Included patient demographics, cardiac function status, presence of pericardial effusion, medical history, and findings from computed tomography angiography (CTA), including the presence of malperfusion syndrome (MPS). Preoperative severity was assessed using the German Registry for Acute Aortic Dissection Type A (GERAADA) score, calculated as follows: Total Score = Age (years) + CPR (0 or 18) + Shock (0 or 10) + (Number of malperfusion sites × 7) + Creatinine >1.5 mg/dL (0 or 4) + Root involvement (0 or 3).
2. To evaluate the efficiency of transfer and surgery: The time from hospital admission to the initiation of surgery was recorded. For the Emergency Bypass Group, the time from transfer dispatch to admission and from admission to surgery initiation was also documented. To assess the risks associated with transfer: Preoperative deaths occurring between admission and surgery initiation were recorded for both groups. Additionally, in the Emergency Bypass Group, deaths occurring between transfer dispatch and hospital admission were also noted.
3. Surgery details: Included whether the surgery was performed during off-hours (non-working hours) and the surgical approach used, such as hemiarch replacement, total arch replacement, and root procedures. Root procedures encompassed commissural suspension, Bentall procedure, David procedure, and Wheat procedure. Additional data included whether concomitant procedures were performed for other structural cardiovascular diseases, coronary artery bypass grafting (CABG), or interventions on aortic branch vessels. Operation time, cardiopulmonary bypass duration, circulatory arrest duration, and lower body circulatory arrest time were recorded. Redo surgeries (defined as failure to complete the initial procedure, requiring suture removal and re-anastomosis of the vascular structure) and re-thoracotomies for hemostasis were also documented.
4. Postoperative outcomes: Included mechanical ventilation duration, reintubation rate, incidence of new-onset stroke, acute kidney injury (AKI), 30-day mortality (including patients discharged against medical advice), in-hospital mortality, and total length of hospital stay.
5. Follow-up: Given the higher proportion of total arch replacement procedures in the Emergency Bypass Group, follow-up outcome measures included all-cause mortality and reintervention rates involving the ascending aorta, aortic arch, thoracoabdominal aorta, or aortic branch vessels. Deaths and reinterventions during the follow-up period were recorded.

### Transfer protocols

#### Routine Referral Group

Upon confirmation of TAAD via aortic CTA at the referring local hospital (which lacked TAAD surgical capabilities), patients were conventionally transferred to the ED of our hospital. Standard procedures included urgent specialist consultation, hospital admission, and subsequent surgery. Postoperative Care: All patients were jointly managed by the surgical and dedicated ICU teams until clinically stable.

#### Emergency Bypass Group

**Figure 1:**
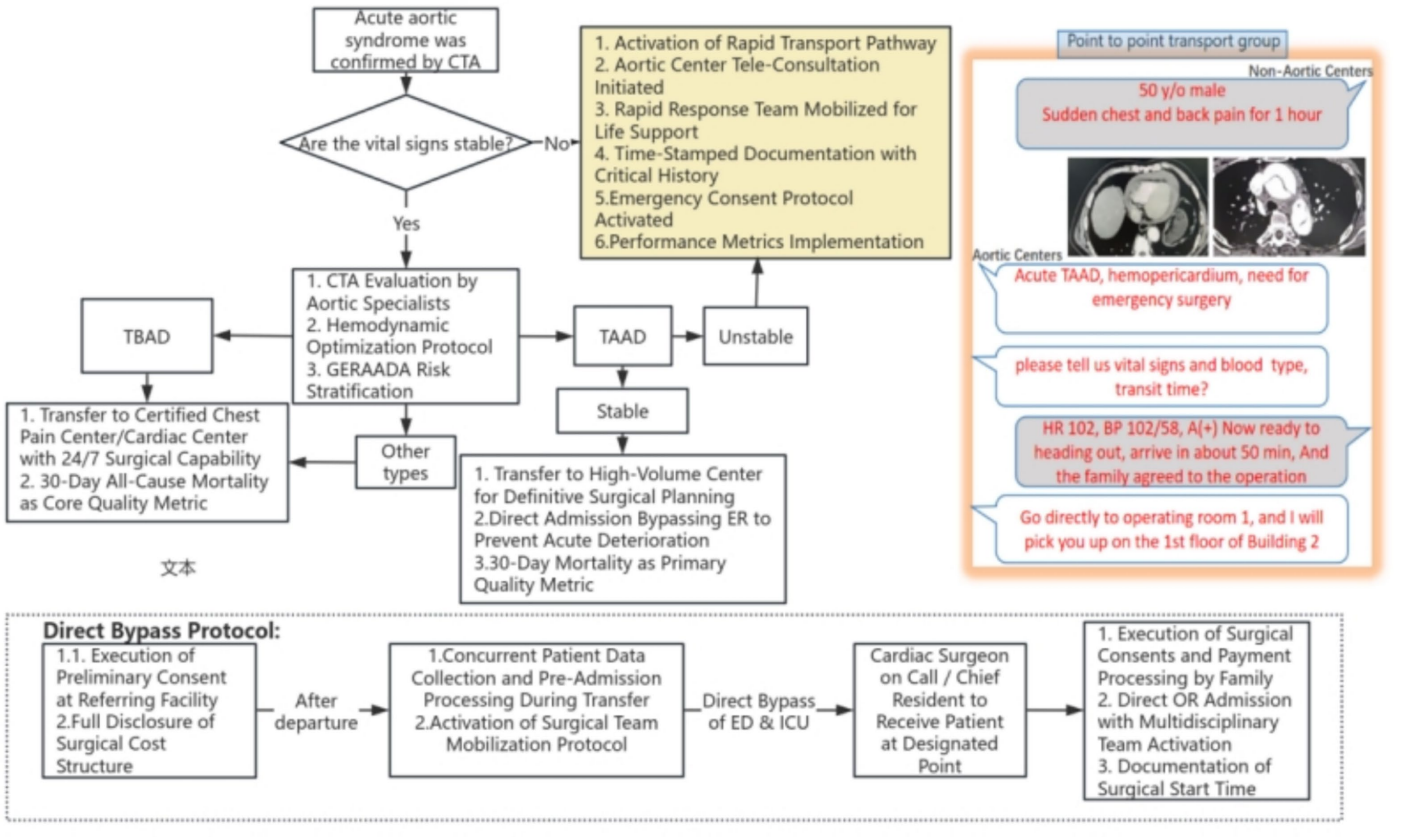
Flowchart of direct transfer and rapid rescue pathway for TAAD.

Initiated in April 2019, this point-to-point bypass protocol was developed based on our chest pain center model, drawing on its transfer experience and collaborating with networked hospitals within a ≤ 3 h transport radius (Figure 1). *Operational Principle:* While “time is life” in critical TAAD cases, rapid transfer must not compromise patient safety. The protocol prioritized minimizing delays while maintaining stable baseline hemodynamics.

Key components:

1. 24/7 “Aortic Dissection Hotline”: Referring hospitals with confirmed TAAD (via CTA) contacted our aortic center’s surgical team directly through a dedicated hotline.
2. Multidisciplinary Thoracic Aortic Surgery Program (TASP): A specialized team at the referring hospital, consisting of emergency physicians, cardiologists, and radiologists. TASP was responsible for:

- Rapid imaging evaluation and standardized hemodynamic management post-diagnosis to reduce early mortality risk (18, 19).
- Real-time transmission of vital signs, CT, and ultrasound images to our center via a telehealth platform for remote guidance during transfer.

3. Bypass Pathway:

- Following remote CTA/clinical review by our aortic team, patients were transported directly to the aortic center by ambulance, bypassing the ED.
- Upon arrival, an aortic specialist assumed care, triaging patients either to the intensive care unit (ICU) for stabilization or directly to the operating room, bypassing both the ED and ICU when appropriate.

4. Surgical Team Structure:

- Two experienced aortic surgeons alternated weekly on-call shifts, with flexible coverage when needed.
- Each shift was staffed by a multidisciplinary team (assistants, perfusionists, anesthesiologists, intensivists) experienced in performing complex aortic surgeries.

5. Postoperative Care: All patients were jointly managed by the surgical and dedicated ICU teams until they were stable enough for transfer back to the referring hospitals or for discharge home.

### Surgical method

The surgical procedure was consistent across the patient cohort and was performed in accordance with previously established protocols (20). Cardiopulmonary bypass (CPB) was initiated via cannulation of the right atrium, femoral artery, left common carotid artery, right axillary artery, or innominate artery. Depending on the extent of aortic dissection and preoperative patient characteristics, the following procedures were performed: total arch replacement, hemiarch replacement, ascending aortic replacement, comprehensive root procedure, or the frozen elephant trunk technique.

### Statistical methods

#### Data processing and basic statistical analysis

Data management and statistical analyses were performed using IBM SPSS Statistics version 22.0 (IBM Corp., Armonk, NY, United States). Normally distributed continuous variables (e.g., age, BMI, total cardiopulmonary bypass time, aortic clamping time, GERAADA score) were expressed as mean ± standard deviation (Mean ± SD). Intergroup comparisons were conducted using independent samples *t*-tests. Non-normally distributed continuous variables (e.g., low-flow time, door-to-incision time, hospital stay) were expressed as median and interquartile range [M (Q1, Q3)]. Intergroup comparisons for these variables were performed using the Mann–Whitney U test (Wilcoxon rank-sum test). Categorical variables were analyzed using Fisher’s exact test (when expected cell counts were <5) or Pearson’s χ^2^ test (for all other cases). A two-sided *p*-value <0.05 was considered statistically significant.

#### Propensity score matching

Propensity scores were estimated using logistic regression models incorporating the following potential confounding factors: history of cardiac surgery, moderate-to-severe pericardial effusion, multi-organ malperfusion syndrome (MPS), diabetes mellitus, GERAADA score, preoperative stroke, total arch replacement, root surgery, prior myocardial infarction/percutaneous coronary intervention, and chronic obstructive pulmonary disease (COPD). Matching method: 1:1 nearest-neighbor matching without replacement, using a caliper width of 0.1 standard deviations. Balance assessment: Standardized mean differences (SMDs) were calculated to assess covariate balance between groups.

#### Survival analysis

Survival probabilities were estimated using the Kaplan–Meier method. Intergroup differences in overall survival and freedom from aortic reintervention were compared using the log-rank test.

#### Multivariate regression modeling

A multivariable Cox proportional hazards regression model was constructed to identify predictors of in-hospital all-cause mortality. Covariates with a univariate *p*-value <0.2 were included in the final model.

## Results

### Preoperative patient characteristics

**Figure 2:**
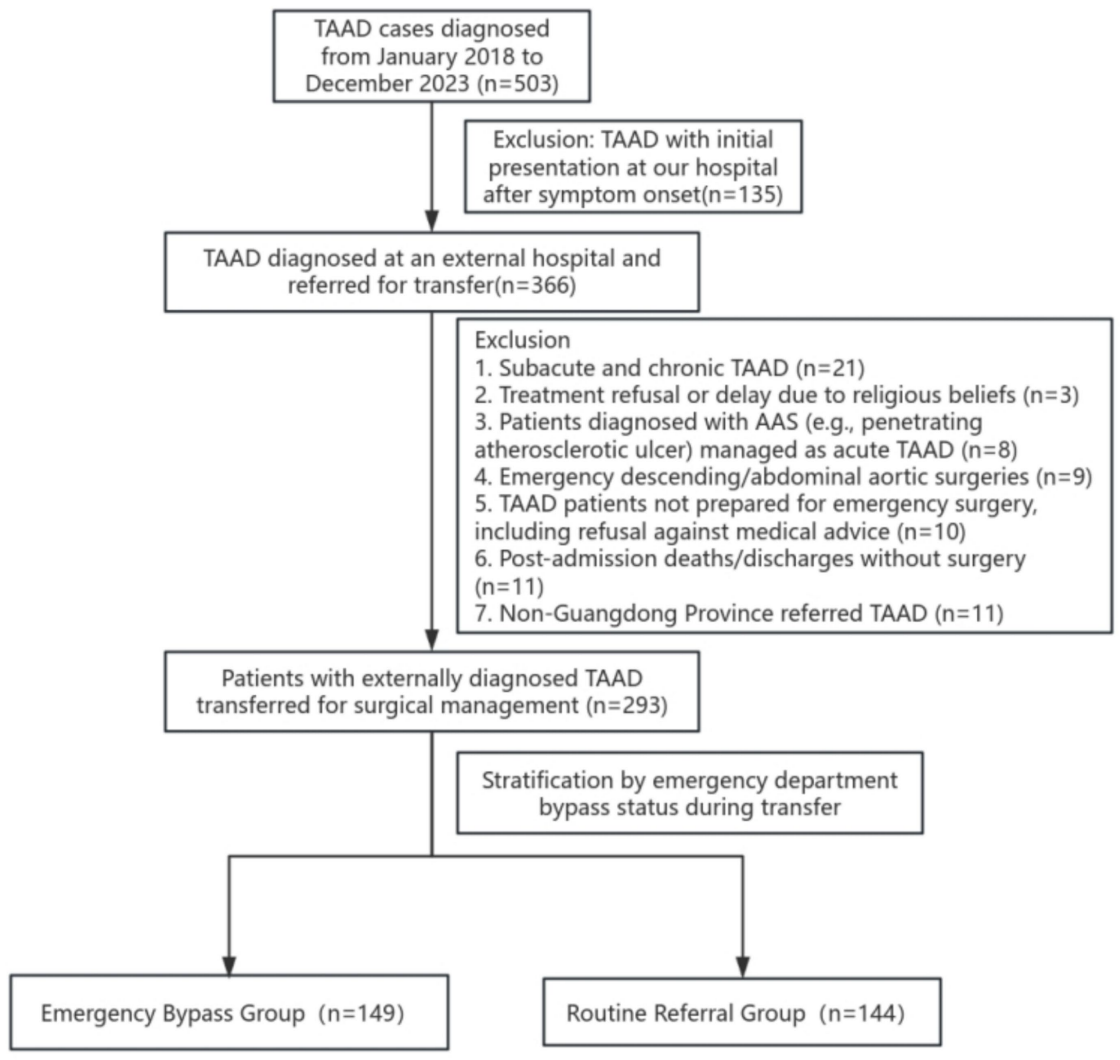
Flowchart of the case screening for the study.

Between January 2018 and December 2023, 505 patients were diagnosed with TAAD. After excluding 135 patients who were initially managed at our center, 370 cases referred from external hospitals were reviewed. Following the exclusion of 73 patients (due to contraindications for surgery, refusal of intervention, or atypical referral pathways), a final cohort of 297 surgically treated patients was identified. Based on referral protocols, patients were stratified into two groups: Emergency Bypass Group (EBG): Patients transferred via the point-to-center ED bypass protocol implemented after April 2019 (*n* = 149). Routine Referral Group (RRG): Patients managed through conventional ED referral pathways (*n* = 144). The study enrollment flowchart is shown in Figure 2.

#### Preoperative mortality

Without excluding in-hospital deaths occurring prior to surgery, the RRG group experienced 8 deaths between admission and surgical intervention, while the EBG group had 9 deaths from the time of ambulance dispatch to surgery (including 3 post-admission but preoperative deaths). No statistically significant difference in preoperative mortality was observed between the two groups (*p* > 0.05) (Table 1).

**Table 1:** Patient demographics and perioperative characteristics.

Demographics/characteristics	Unmatched		*P*-value	SMD	Matched		*P*-value	SMD
	Routine Referral Group (*n* = 144)	Emergency Bypass Group (*n* = 149)			Routine Referral Group (*n* = 112)	Emergency Bypass Group (*n* = 112)		
Female, *n* (%)	24 (16.667)	22 (14.765)	0.655	0.052	18 (16.071)	15 (13.393)	0.572	0.076
Age, Mean ± SD	52.819 ± 11.743	52.148 ± 11.564	0.622	0.058	52.938 ± 11.453	51.464 ± 11.831	0.345	0.127
BMI, Mean ± SD	25.977 ± 3.994	26.290 ± 3.731	0.489	0.081	25.960 ± 3.870	26.358 ± 3.806	0.438	0.104
Previous cardiac surgery, *n* (%)	2 (1.389)	9 (6.040)	0.036	0.248	2 (1.786)	2 (1.786)	1	0
Diabetes, *n* (%)	12 (8.333)	7 (4.698)	0.206	0.148	10 (8.929)	7 (6.250)	0.449	0.101
Door-to-incision time, M (Q1, Q3)	8.000 (5.000, 12.000)	4.000 (3.000, 6.000)	<0.001	1.071	8.000 (5.000, 12.250)	4.500 (3.000, 6.250)	<0.001	1.206
Non-business hours surgery *n* (%)	47 (32.639)	61 (40.940)	0.141	0.173	40 (35.714)	49 (43.750)	0.219	0.165
GERAADA, Mean ± SD	59.583 ± 11.400	61.020 ± 11.588	0.286	0.125	59.812 ± 11.287	60.192 ± 11.704	0.805	0.033
Current smoker, *n* (%)	77 (53.472)	70 (46.980)	0.267	0.13	63 (56.250)	53 (47.321)	0.181	0.179
Previous stroke, *n* (%)	6 (4.167)	4 (2.685)	0.706	0.082	4 (3.571)	4 (3.571)	1	0
History of hypertension, *n* (%)	117 (81.250)	125 (83.893)	0.551	0.07	86 (76.786)	93 (83.036)	0.243	0.156
Ejection fraction <50%, *n* (%)	7 (4.861)	11 (7.383)	0.049	0.105	6 (5.357)	6 (5.357)	1	0
Moderate to large pericardial effusion, *n* (%)	16 (11.111)	31 (20.805)	0.024	0.267	13 (11.607)	9 (8.036)	0.369	0.12
MPS, *n* (%)	22 (15.278)	37 (24.832)	0.041	0.24	17 (15.179)	15 (13.393)	0.703	0.051
Previous myocardial infarction, *n* (%)	5 (3.472)	6 (4.027)	0.803	0.029	4 (3.571)	3 (2.679)	1	0.051
Creatinine >1.5 mg/dL, *n* (%)	34 (23.611)	37 (24.832)	0.807	0.029	29 (25.893)	23 (20.536)	0.342	0.127
Ventilated preoperatively, *n* (%)	37 (25.694)	54 (36.242)	0.051	0.23	34 (30.357)	39 (34.821)	0.476	0.095
Hyperlipidemia, *n* (%)	29 (20.139)	34 (22.819)	0.577	0.065	24 (21.429)	24 (21.429)	1	0
COPD, *n* (%)	9 (6.250)	8 (5.369)	0.747	0.038	3 (2.679)	5 (4.464)	0.719	0.096
CPB time, Mean ± SD	122.236 ± 18.662	119.517 ± 15.459	0.175	0.159	122.205 ± 17.728	119.714 ± 15.499	0.264	0.15
Cross-clamp time, Mean ± SD	65.458 ± 11.585	64.933 ± 11.000	0.691	0.047	65.714 ± 11.889	64.732 ± 10.780	0.518	0.087
HCA time, M (Q1, Q3)	16.000 (13.000, 19.000)	15.000 (14.000, 17.000)	0.162	0.116	16.000 (13.000, 18.000)	15.000 (14.000, 17.000)	0.449	0.149
Total aortic arch replacement, *n* (%)	121 (84.028)	136 (91.275)	0.059	0.222	100 (89.286)	103 (91.964)	0.492	0.092
Aortic root surgery, *n* (%)	43 (29.861)	44 (29.530)	0.951	0.007	32 (28.571)	29 (25.893)	0.653	0.06
Combine other surgeries, *n* (%)	10 (6.944)	8 (5.369)	0.575	0.066	8 (7.143)	7 (6.250)	0.789	0.036
Re-do, *n* (%)	6 (4.167)	3 (2.013)	0.466	0.125	5 (4.464)	3 (2.679)	0.719	0.096
Perform vascular branch intervention, *n* (%)	9 (6.250)	8 (5.369)	0.747	0.038	7 (6.250)	6 (5.357)	0.775	0.038
CABG, *n* (%)	9 (6.250)	12 (8.054)	0.55	0.07	8 (7.143)	10 (8.929)	0.623	0.066

*P-value*<*0.05. OP time, Operation time; CPB time, cardiopulmonary bypass time; CA time, Cardiac arrest time; HCA, hypothermic circulatory arrest; Re-do, The surgery failed to be completed successfully, and the sutures had to be removed to redo the vascular anastomosis; COPD, chronic obstructive pulmonary disease; CABG, coronary artery bypass grafting; MPS, malperfusion syndrome.

#### Baseline characteristics

The two groups showed comparable baseline characteristics in terms of age, sex, smoking status, renal dysfunction, chronic obstructive pulmonary disease (COPD), hypertension, prior myocardial infarction, GERAADA score, and history of stroke (all *p* > 0.05). Under the guidance of aortic specialists, the EBG group demonstrated a higher rate of preoperative mechanical ventilation (25.7% vs. 36.2%, *p* = 0.051, HR = 1.644, 95% CI: 0.996–2.714). However, the EBG group had significantly higher proportions of the following conditions: left ventricular ejection fraction (EF) < 50%, multi-organ malperfusion syndrome (MPS), pericardial tamponade, and prior cardiac surgery (all *p* < 0.05). To address these confounding imbalances, 1:1 propensity score matching (PSM) was performed using nearest-neighbor matching (caliper width: 0.1, no replacement). The post-matching analysis included 112 patients in each group, with all covariates achieving acceptable balance (standardized mean differences <0.1 for all variables).

### Intraoperative and early postoperative outcomes

Compared to the control group, the direct bypass group demonstrated a significantly shorter door-to-surgery time, both before and after propensity score matching. Surgical complexity was notably higher in the bypass group, as indicated by the greater proportion of total arch replacement procedures (84.0% vs. 91.3%, *p* = 0.059, HR = 1.989, 95% CI: 0.965–4.097) approaching statistical significance (Table 2).

**Table 2:** Postoperative complications.

Complication	Unmatched		*P*-value	SMD	Matched		*P*-value	SMD
	Routine Referral Group (*n* = 144)	Emergency Bypass Group (*n* = 149)			Routine Referral Group (*n* = 112)	Emergency Bypass Group (*n* = 112)		
Re-exploration for hemostasis, *n* (%)	12 (8.333)	7 (4.698)	0.206	0.148	5 (4.464)	5 (4.464)	1	0
AKI, *n* (%)	43 (29.861)	34 (22.819)	0.171	0.16	34 (30.357)	23 (20.536)	0.092	0.227
Reintubation, *n* (%)	12 (8.333)	9 (6.040)	0.447	0.089	11 (9.821)	8 (7.143)	0.472	0.096
Mechanical ventilation time >48 h, *n* (%)	80 (55.556)	78 (52.349)	0.582	0.064	62 (55.357)	55 (49.107)	0.349	0.125
New stroke, *n* (%)	10 (6.944)	6 (4.027)	0.272	0.128	7 (6.250)	4 (3.571)	0.354	0.124
30-d mortality, *n* (%)	19 (13.194)	12 (8.054)	0.153	0.167	14 (12.500)	5 (4.464)	0.031	0.291
In-hospital mortality, *n* (%)	27 (18.750)	16 (10.738)	0.053	0.227	17 (15.179)	8 (7.143)	0.056	0.257
LOS (length of stay), M (Q1, Q3)	16.000 (14.000, 19.000)	17.000 (15.000, 21.000)	0.101	0.116	16.000 (14.000, 19.000)	17.000 (15.000, 21.000)	0.003	0.32

*P-value <0.05.

Surgical parameters including operative techniques, cardiopulmonary bypass duration, circulatory arrest time, reoperation rates, and concurrent procedures did not differ significantly between the two groups. Likewise, early postoperative outcomes such as bleeding/re-exploration rate (*n*, %), incidence of new-onset stroke (*n*, %), mechanical ventilation exceeding 48 h, and acute kidney injury (AKI, defined as a > 50% increase from baseline creatinine) showed no statistically significant differences.

#### Mortality outcomes

The bypass group demonstrated significantly lower in-hospital mortality both before (18.75% vs. 10.74%, *p* = 0.053, HR = 1.918, 95% CI: 0.985–3.735) and after propensity score matching (15.2% vs. 7.1%, *p* = 0.056, HR = 2.326, 95% CI: 0.960–5.638). While no statistically significant difference in 30-day mortality was observed before matching, post-matching analysis revealed a significant reduction in the bypass group (12.5% vs. 4.46%, *p* = 0.031).

### Supplementary outcomes

#### 1. Kaplan–Meier survival analysis

**Figure 3:**
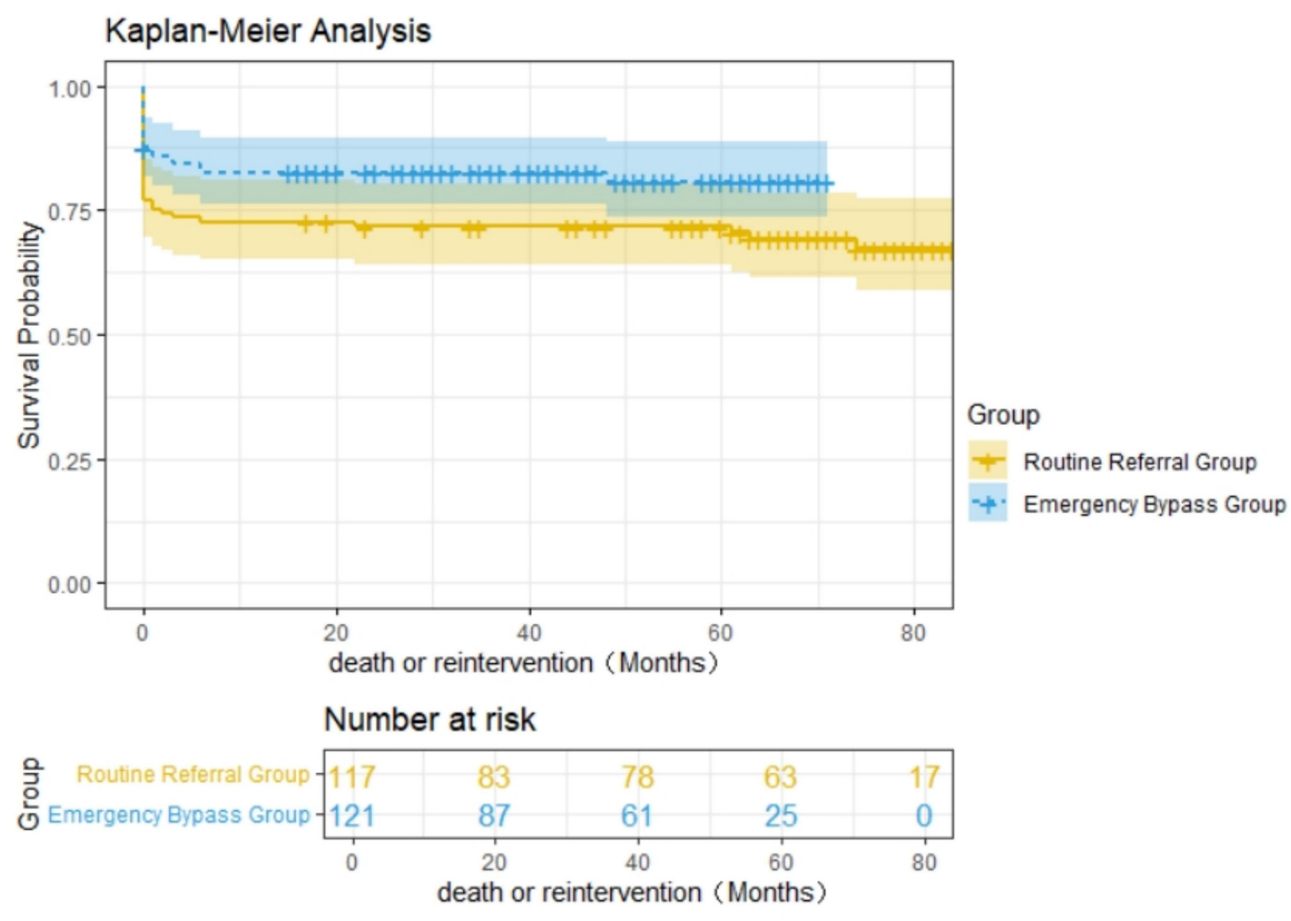
The Kaplan–Meier survival curve showing the impact of the service on survival or reintervention.

Single-factor analysis of follow-up survival data demonstrated significant improvement in long-term survival/freedom from reintervention in the Emergency Bypass Group compared to the Routine Referral Group (log-rank *p* = 0.049; Figure 3).

#### 2. Cox multivariable analysis of in-hospital mortality (pre-matching cohort)

Protective and Risk Factors of mortality included: Reduced left ventricular ejection fraction (EF) (HR = 3.279, 95% CI: 1.14–9.43, *p* = 0.028), Moderate-to-large pericardial effusion (HR = 6.19, 95% CI: 2.59–14.81, *p* < 0.001), Concomitant multi-organ malperfusion (HR = 2.90, 95% CI: 1.22–6.92, *p* = 0.016).

Total aortic arch replacement emerged as a protective factor against mortality (HR = 0.387, 95% CI: 0.159–0.943, *p* = 0.037). Conventional referral pathways (Routine Referral Group) independently increased overall mortality risk (HR = 4.546, 95% CI: 1.721–12.004, *p* = 0.002), as detailed in Table 3.

**Table 3:** Univariable and multivariable analyses of prognostic factors for overall survival.

Factor	Univariable *P*-value	Multivariable *P*-value	Hazard ratio	95% CI lower	95% CI upper
Previous cardiac surgery	0.635				
Current smoker	0.873				
Non-business hours surgery	0.751				
BMI	0.095	0.595			
Female	0.034	0.146			
Age	<0.001	0.229			
Previous stroke	<0.001	0.310			
Creatinine >1.5 mg/dL	0.177	0.975			
Ventilated preoperatively	0.042	0.887			
Previous myocardial infarction	<0.001	0.316			
Perform vascular branch intervention	0.003	0.112			
CABG	<0.001	0.197			
Aortic root surgery	0.003	0.114			
Hyperlipidemia	0.042	0.800			
Diabetes	0.163	0.595			
Re-do	<0.001	0.785			
COPD	<0.001	0.051	2.630	0.995	6.951
Routine Referral	0.100	0.002	4.546	1.721	12.004
Aortic arch replacement	<0.001	0.037	0.387	0.159	0.943
Ejection fraction <50%	<0.001	0.028	3.279	1.140	9.433
Moderate to large pericardial effusion	<0.001	<0.001	6.191	2.587	14.815
MPS	<0.001	0.016	2.901	1.217	6.916

*P-value <0.05.

## Discussion

The development of Chest Pain Centers (CPCs) has significantly shortened the time from symptom onset to diagnosis for TAAD (21). However, some diagnosing hospitals may contact multiple aortic centers simultaneously for referrals. Given the extremely poor natural prognosis of TAAD after onset, this uncertain transfer process can delay referral and surgery, thereby increasing mortality risk. Therefore, managing post-diagnosis referral and surgical timing is crucial to improving TAAD outcomes. Optimizing referral models and establishing regionalized “TAAD Centers” is essential to reduce mortality.

To address prolonged transfer delays, Igor et al. (22) analyzed 199 TAAD cases from January 2010 to December 2021, dividing them into two groups based on the implementation of regionalized surgical services in 2016: Group 1 (2010–2015, *n* = 90) and Group 2 (2016–2021, *n* = 109). Following regionalization, mortality decreased from 13 to 4%, time from diagnosis to surgery was reduced from 210 to 160 min, and the incidence of preoperative cardiac tamponade and shock decreased. Similarly, Ricky et al. (17) prospectively compared outcomes before (2003–2011) and after (2011–2017) implementing a 24 h “aortic dissection hotline” serving 2.4 million people in Northwest London. This system involved a rotating on-call team of seven cardiac surgeons from three tertiary hospitals (Royal Brompton, Harefield, Hammersmith), supported by perfusionists, anesthesiologists, and intensivists. Analysis of 227 surgical TAAD cases showed a significant reduction in 30-day mortality from 20 to 8%. In China, where prehospital emergency care for TAAD remains suboptimal, Fuwai Hospital established a Beijing-centered, 300-kilometer radius regional acute aortic dissection network with 12 collaborating hospitals in September 2022 (23). Our institution pioneered the national Chest Pain Center and launched Guangdong Province’s first 24 h “aortic dissection hotline” in 2019, creating a point-to-point transfer system for acute aortic syndromes. This multidisciplinary Thoracic Aortic Surgery Program (TASP), conceptualized by Andersen (24) and Antoniou (25), integrates emergency physicians and cardiologists from referring hospitals with aortic center specialists. TASP enables rapid diagnosis and treatment decisions even when on-site aortic teams are unavailable, optimizing transfer efficiency while reducing surgical team workload. Compared to conventional transfers, this model reduced median diagnosis-to-surgery time and lowered in-hospital mortality (18.75% vs. 10.74%, *p* = 0.053), with a significant decrease in 30-day mortality after propensity matching (12.5% vs. 4.46%, *p* = 0.031), effectively mitigating both prehospital transfer delays and in-hospital surgical preparation delays.

TAAD patients can be categorized as stable or unstable (critical). Rapid transfer of unstable TAAD patients is lifesaving and essential for managing acute complications, while delayed surgery at high-volume centers benefits most stable cases. Berretta et al. (3) defined unstable TAAD by the presence of preoperative cardiac tamponade, shock, congestive heart failure, cerebrovascular accident, stroke, coma, myocardial ischemia/infarction, acute renal failure, or mesenteric ischemia/infarction. Both Berretta and Gasser reported that more than half of surgically treated TAAD patients were unstable at the time of surgery (3, 4). Analysis of 4,428 patients from the IRAD registry (1995–2013) showed that unstable patients had shorter symptom-to-surgery intervals (3.4 vs. 5.0 h in stable patients), indicating that shorter time to surgery reflects higher risk (3). Gasser et al. (4) stratified 283 TAAD patients (median symptom-to-incision time: 6.9 h) into three groups: <4 h, 4–10 h, and >10 h. The <4 h group showed higher preoperative severity including neurologic deficits, tamponade, and CPR rates and higher postoperative mortality, emphasizing the urgency of timely diagnosis and surgery, especially for unstable cases. Gasser also noted many unstable TAAD patients die before reaching the operating room, underscoring the need to minimize diagnosis-to-surgery time to improve survival (4). Results from a 2003–2020 nationwide, big-data retrospective study led by Professor McClure in Canada revealed that TAAD patients requiring transfer for surgical evaluation had twice the overall mortality risk compared to those undergoing direct surgery without transfer (12). Regional rapid transfer protocols significantly reduce diagnosis-to-surgery delays, preventing procedural delays and securing surgical opportunities for unstable TAAD patients. In this study, the point-of-care ED bypass pathway greatly shortened diagnosis-to-surgery time for critically ill transfers, allowing direct bypass of ED/ICU for select unstable patients. Compared to Fuwai Hospital’s reported 13 h door-to-surgery time, this protocol enabled immediate surgery for unstable cases. The bypass group had a significantly shorter median door-to-surgery time (4 vs. 8 h, *p* < 0.01), but higher clinical severity (pericardial effusion, reduced ejection fraction, multi-organ malperfusion syndrome [MPS], prior cardiac surgery; all *p* < 0.05). This can be attributed to the specialized point-to-point support provided to local hospitals, which streamlined the referral process, enhanced the expertise in medical management, and established an in-hospital green channel for direct transfer to the operating room. These systemic improvements collectively increased the proportion of critical TAAD patients who became eligible for surgical intervention. Propensity score matching (1:1) was applied to reduce confounding. Pre-matching Cox regression identified conventional transfer, pericardial effusion, reduced ejection fraction, and MPS as independent mortality risk factors. Excluding pre-surgical deaths likely underestimated the survival benefit; including them would further highlight the pathway’s effectiveness. Post-matching analysis confirmed improved survival outcomes, reinforcing the model’s value in optimizing TAAD management.

TAAD patients require careful balancing of the risks and benefits associated with aortic arch reconstruction. For hemodynamically unstable, older adults, or frail patients, a more conservative approach such as isolated ascending aortic replacement may be preferable. However, at specialized aortic centers, particularly for younger patients, aggressive correction of extensive aortic pathology can be performed safely with favorable outcomes (26–28). In Ricky’s study (15), the proportion of patients undergoing aortic arch interventions increased following the implementation of a regionalized referral system (11% vs. 20%, *p* = 0.074). Multivariable analysis identified aortic arch surgery as an independent risk factor for poorer prognosis (*p* = 0.00, HR 3.36; 95% CI 1.77–6.4), yet the regionalized referral model still led to significant improvements in overall 30-day mortality (20.3% vs. 8.1%, *p* = 0.01) and long-term survival. In our study using a point-to-point regionalized referral model, surgeons showed better preparation for TAAD patients, reflected in a higher rate of total arch replacement (84.0% vs. 91.3%, *p* = 0.059). Additionally, patients undergoing total arch replacement had better preoperative baseline conditions. Multivariable analysis demonstrated that total arch replacement was protective against mortality (HR = 0.387, 95% CI: 0.159–0.943, *p* = 0.037). Kaplan–Meier analysis showed significant improvements in survival and freedom from reintervention in the experimental group, likely due to the increased use of total arch replacement and routine application of the frozen elephant trunk technique during arch surgery. This approach reduces the need for subsequent interventions on the arch or descending thoracic aorta during follow-up. Although the experimental group presented with more severe conditions, hospital length of stay was significantly reduced both before and after propensity score matching. This is likely because, under the point-to-point referral model, stabilized patients were transferred back to local hospitals for convalescence after surgery at our center. Notably, the proportion of patients with prior cardiac surgery was higher in the experimental group (14% vs. 4%, *p* = 0.011), which may reflect improved inter-hospital communication through the referral system and the preference to transfer technically complex TAAD cases with previous open-heart surgery to high-volume centers.

## Limitations

First, the majority of the RRG group cases were collected between January 2018 and April 2019, while all the EBG group cases were enrolled from April 2019 to December 2023. During this five-year period (2018–2023), significant advancements occurred in surgical techniques, perioperative management, and other relevant aspects. These temporal improvements may serve as independent risk factors affecting postoperative mortality. It is important to note that cases before 2019 were not collected prospectively. Collectively, these factors may compromise the accuracy and reliability of our study findings. Second, excluding patients who declined surgery or died preoperatively may lead to an overestimation of survival rates compared to real-world outcomes. However, the comparison between preoperative deaths during transfer in the experimental group (9 cases) and in-hospital pre-surgical deaths in the control group (8 cases) suggests that the point-to-point transfer model could reduce preoperative mortality, which is a significant advantage. Finally, as a single-center study, these findings require validation through multicenter studies with larger patient cohorts to confirm their generalizability and broader clinical relevance.

## Conclusion

Integrating streamlined prehospital and preoperative services with a specialized aortic team significantly improves outcomes for patients with TAAD. Given the poor natural prognosis of TAAD, the fact that 60% of patients treated at high-volume centers are transferred from external hospitals, and that roughly half present as unstable at surgery, establishing a regionalized point-to-point transfer system in collaboration with the Chest Pain Center is crucial. This emergency department bypass model substantially shortens in-hospital response times, offering critically unstable TAAD patients timely access to life-saving surgery and reducing potential survivorship bias in research. Additionally, this specialized approach lowers postoperative mortality and the need for reintervention. Overall, optimizing and simplifying the TAAD referral pathway in partnership with experienced aortic centers markedly improves patient prognosis.

## Data Availability

The raw data supporting the conclusions of this article will be made available by the authors, without undue reservation.
